# Sexual differences in phenotypical predictors of floating status: body condition influences male but not female reproductive status in a wild passerine

**DOI:** 10.1007/s00442-022-05180-1

**Published:** 2022-05-12

**Authors:** Iraida Redondo, Lorenzo Pérez-Rodríguez, Raquel Monclús, Jaime Muriel, Diego Gil

**Affiliations:** 1 Departamento de Ecología Evolutiva, Museo Nacional de Ciencias Naturales (MNCN), CSIC, José Gutiérrez Abascal 2, 28006 Madrid, Spain; 2 Instituto de Investigación en Recursos Cinegéticos (IREC), CSIC-UCLM-JCCM, Ronda de Toledo 12, 13005 Ciudad Real, Spain; 3 Laboratoire d’Ethologie Expérimentale et Comparée UR 4443, Université Sorbonne Paris Nord, 93430 Villetaneuse, France; 4 Department of Zoology, Faculty of Sciences, University of Granada, 18071 Granada, Spain

**Keywords:** Floating, Starling, Reproductive-status, Nonbreeders, Early-conditions

## Abstract

Floaters constitute the sexually mature but non-breeding part of populations. Despite being ubiquitous in most species, knowledge about floaters is scarce. Ignoring this significant number of individuals may strongly bias our understanding of population dynamics and sexual selection processes. We used the spotless starling (*Sturnus unicolor*) to examine whether phenotypical and non-phenotypical variables from early and adult life predict reproductive status, focusing on the earliest age at which most individuals start to breed, when the percentage of floaters is the highest. We compiled data from a long-term study involving eight female and seven male cohorts of individuals PIT-tagged at birth. We compared a suite of nestling (condition, hatching date and brood size) and adult variables (condition, size and ornamentation) between floaters and breeders. We found that adult and nestling body condition strongly and positively influenced the likelihood of breeding in males. Path analysis revealed that male reproductive status could only be predicted by considering nestling body condition—the influence of this variable superseded adult body condition. Female reproductive status was only negatively associated with hatching date. Ornamentation was not associated with reproductive status in any of the sexes, although path analyses revealed that body condition was positively associated with throat feather length. We conclude that predictors of reproductive status are sex-specific in the spotless starling, suggesting an important role of body condition in access to breeding resources in males. Our results also highlight the long-term influence of early life on life trajectories and their potential implications on floating status.

**Supplementary Information:**

The online version contains supplementary material available at 10.1007/s00442-022-05180-1.

## Introduction

Breeding resources (e.g. nesting sites, breeding mates) are finite, limiting the number of individuals that can reproduce in a breeding season (Newton 1992). Thus, two categories of individuals can be distinguished: (1) breeders (also denominated ‘owners’ and ‘territory holders’ in the literature): birds that have succeeded in acquiring those resources to breed, and (2) floaters: a surplus of birds that coexist with breeding individuals and that lack the required resources to reproduce (Brown 1969).

The existence of floaters in avian populations can be explained by two not mutually exclusive hypotheses. The first hypothesis considers that floaters are a surplus of lower quality individuals lacking the abilities to get breeding resources during competition (Smith and Arcese 1989; Lozano 1994). The second hypothesis sees floating behaviour as an alternative reproductive strategy in which individuals delay reproduction to enhance their fitness later in life (e.g. by getting higher quality areas to breed; Zack and Stutchbury 1992; Bruinzeel and van de Pol 2004). Reviews on this topic underline the scarcity of true alternative reproductive strategies in birds (Johnson et al. 2000) and conclude that most data show that floating behaviour is a conditional strategy, with floaters waiting for breeding opportunities (e.g. vacancies) to arise (Moreno 2016). Independently of whether floating status is a constraint or a ‘decision’, little is known about the phenotypical traits that may influence reproductive status. This gap of information is partly caused by floaters’ secretive behaviour, which ultimately biases our knowledge towards more conspicuous (i.e. breeders) individuals (Moreno 2016).

In avian species that rely on discrete and limited resources for breeding (e.g. cavity-nesting species), differences in competitiveness are expected to strongly determine whether an individual becomes a breeder or a floater, as competition for nesting sites in these species is intense (Newton 1998). Differences in competitive ability are primarily influenced by body size or body condition, as bigger and fitter individuals have a better capability of acquiring and holding resources (Searcy 1979). Besides its influence on competition, low body condition may increase the probability of individuals delaying reproduction to maximize fitness in the future, but this is more likely to occur in long-lived species (Becker and Bradley 2007). Several studies in the past have examined if morphological differences between breeders and floaters determine reproductive status, reporting mixed results: while some of these studies did not find differences in body size or body condition (Shutler and Weatherhead 1991; Peer et al. 2000; Sergio et al. 2009), others found floaters to be smaller or in worse condition than breeders (Lozano 1994; Sandell and Diemer 1999; Emlen and Wrege 2004). In addition to body condition or body size, studies comparing floater and breeder morphology often incorporated ornamental characteristics in their analyses. Ornaments are considered reliable indicators of phenotypic and genetic quality, playing significant roles in social and sexual signalling (Espmark et al. 2000; Andersson 1994). Indeed, empirical studies provide compelling evidence that breeders tend to display bigger or more elaborated ornaments than floaters (Johnson et al. 2000; Velando et al. 2001; Pryke and Andersson 2003; Emlen and Wrege 2004).

Depending on the species’ ecology and breeding system, differences in phenotype (i.e. condition, size, ornamentation) may be more relevant for one sex than for the other. In the case of polygynous species or species where mainly males compete over resources, the sex ratio of the floating population is expected to be male-biased (Moreno 2016). Thus, differences in phenotype are expected to be more marked in males than in females. However, females are also present as floaters (though in smaller proportions; Moreno 2016) but have received less attention than males.

Many adult traits associated with breeding status are influenced by conditions experienced during development. Early life conditions, which include a wide variety of factors (e.g. parental quality, food quality and availability, temperature, sibling competition, etc.), have the potential to greatly impact morphological and life-history traits in the short and long term (Lindström 1999; Metcalfe and Monaghan 2001; Naguib and Gil 2005) by influencing nestling morphology and physiology as well as the subsequent adult phenotype (Boag 1987; Saino et al. 2018). Thus, developmental conditions may result in variation of key life-history traits such as survival, recruitment, and reproductive success (Cam and Aubry 2011). For example, individuals developing under favourable early life conditions (also referred to as ‘silver spoon’ effect) benefit from a higher fledgling survival and recruitment by reaching a bigger size or greater body weight (Suedkamp-Wells et al. 2007; Becker and Bradley 2007; Vitz and Rodewald 2011). To our knowledge, there are no studies examining how early life may influence floating status, mainly due to the scarcity of long-term studies and the difficulty associated with the monitoring of marked floaters.

The spotless starling (*Sturnus unicolor*, Temm. 1820) is a semi-colonial secondary-cavity nester that readily occupies nest-boxes. High local density and facultative polygyny lead to strong competition over nesting sites, resulting in a surplus of male and female non-breeding birds (i.e. floaters) that coexists with breeders. In this species, floaters are usually observed intruding nest-boxes already occupied by conspecifics and are also detected by the fast occupation of nest-boxes provided even in the middle of the breeding season (Veiga et al. 2012; D. Gil, unpublished observations). The spotless starling is a relatively long-lived bird (Veiga and Polo 2011) in comparison with other passerines, and this sets the stage for delayed reproduction tactics and successful floater strategies. Both sexes reach their sexual maturity as 1-year-olds. However, in contrast to females, males rarely breed as 1-year olds, most recruiting into the breeding population as 2- or 3-year-old adults. This delay in reproduction together with limiting breeding sites creates a high-competition scenario for reproduction in males, providing a good framework to investigate phenotypical differences between floaters and breeders. In this species, fights between females are not uncommon (pers. obs.), suggesting that reproductive status in females may also be determined by phenotypical differences.

In this work, we used data collected from long-term monitoring (2012–2020) of a wild colony of spotless starlings. We gathered longitudinal information of female and male spotless starlings belonging to 8 and 7 different cohorts respectively and followed them until the age at which males or females typically start breeding. By marking individuals with passive integrated transponder tags (hereafter, PIT-tags) we took advantage of their frequent intruding behaviour, deploying automatized PIT-tag readers in nest-boxes during reproduction to detect birds that would have gone unnoticed otherwise. This intensive monitoring fieldwork carried out from 2012 until 2020 allowed us to assemble a large sample size of individuals of known exact age and sex.

This study examines if a suite of morphological traits commonly associated with resource acquisition can determine the reproductive status (floater vs. breeder), both in male and female spotless starlings facing their first reproductive attempt. Specifically, we examined whether body condition, body size and ornament size were related to male and female reproductive status. In the case of females, we also included a measure of delayed plumage maturation, plumage spottiness, that may be relevant in competition for resources. We predicted that adult individuals in better body condition and with more elaborate ornamentation would be more likely to become breeders and that the effect would be more pronounced in males than females. Lastly, as all individuals involved in this study were monitored since their birth, we also investigated if several variables related to early life (nestling condition, hatching date and brood size) had long-term effects on adult reproductive status, and to what extent the effect of these traits was mediated by adult phenotype. Since early conditions can have carry-over effects, we predicted nestling body condition to indirectly influence adult reproductive status, with nestlings in better body condition having a higher likelihood of becoming breeders. Late hatching date (because of worsening of conditions) and larger brood size (because of stronger competition) are known to negatively affect quality, thus we predicted that spotless starlings reared later in the season and in larger broods would have a lower likelihood of becoming breeders as 1-year and 2-year-old birds (females and males, respectively).

## Materials and methods

### Study area

We performed a long-term monitoring effort (2012–2020) of a spotless starling colony located in central Spain (Soto del Real, Madrid). The study area is characterized by an open woodland of oaks (*Quercus pyrenaica*) and ashes (*Fraxinus angustifolia*) and the presence of grazing livestock. In the study area, there are 246 nest-boxes that we monitor during the whole breeding season (March-July).

### Study species

The spotless starling (hereafter, ‘starling’) is a non-migratory passerine species that is distributed in the western Mediterranean. It is a strict cavity-nesting species that only defends a small area around the nest-site against conspecifics. Starlings often live in colonies where several nests can be found in proximity. This species shows a facultative polygynous mating system in which males can defend more than one nest-site (Cordero et al. 2001), although high levels of male-male competition reduce polygamy (Celis et al. 2021). Extra-pair paternity shows moderate levels (ca. 19% broods, 7% nestlings) as well as conspecific brood parasitism (21% broods, 7% nestlings). Nest sabotages typically involving egg ejection are very common, in particular in first broods, where ca. 20% of broods are sabotaged before or during incubation (unpublished data). Nest take-overs, sometimes involving infanticide, have also been detected.

Males and females exhibit sexual dimorphism with males being bigger and showing a blueish patch at the base of the beak as well as longer and modified ornamental throat feathers (Hiraldo and Herrera 1974; Online resource 4). The length of these feathers increases with age (Hiraldo and Herrera 1974) and is related to proxies of individual quality (Aparicio et al. 2001; Gil and Culver 2011; Ruiz-Rodríguez et al. 2015). Males conspicuously display them during courtship and singing bouts, which is consistent with social and sexual signalling roles. Female throat feathers are much shorter, but their length also increases with age and is also related to individual breeding output (López-Rull et al. 2007), which is consistent with a quality signalling function in this sex as well. Although the plumage of adults is mostly uniformly black, 1-year-old males usually exhibit a female-like speckled plumage that is replaced by a glossy plain black plumage as 2-year-olds. In the case of females, white spots are numerous and bigger in younger females, although the gradual disappearance of spots with age is highly variable among individuals. Females typically lay two clutches with a modal clutch size of 5 eggs: the first one between mid-April and early May, and the second one at the end of May and the onset of June. Fledglings leave the nest when they are approximately 22 days old.

### Field methodology

For this work, we gathered longitudinal data involving 8 and 7 cohorts of female and male birds respectively (cohorts fledged from 2012 to 2019, with breeding data collected up to 2020), encompassing a total of 10,091 nestlings. Hatching date of nestlings was monitored by visiting the nest-boxes every day after the completion of the incubation period (11–12 days). Nestlings from each cohort were marked with a unique number-coded metal ring and a PIT-tag (ID-100 Trovan Unique Implantable tags, Trovan Ltd, UK) and measured when they were 14 days old. We measured body weight with a digital scale (Ohaus, Model CS200, Pine Brook, New Jersey) to the nearest 0.1 g and tarsus length with a digital calliper (Mitutoyo Absolute, Japan) to the nearest 0.01 mm.

For the analysis, we selected birds marked and measured as nestlings that were captured as adults at the age in which males or females typically start breeding, that is, 1 year after birth in the case of the females, and 2 years later in the case of males (Veiga et al. 2012). This sexual difference in the age of first reproduction was determined by following the breeding trajectory of two fully PIT-tagged nestling cohorts (2016, 2017). From 211 monitored males, only 3% of males were found to breed as 1-year olds, supporting our assumption that male starlings usually start breeding as 2- and 3-year-olds (online resource 1 and online resource 2). In contrast, 33% of females started to breed as 1-year-olds. This difference in the age of first reproduction suggests that competition for breeding resources is higher for males than for females.

Most adults were captured one month before egg-laying. We captured individuals that were sleeping inside the nest-box just before sunrise by blocking the nest entrance, as well as those that visited nest-boxes during the rest of the morning (0800-1200) by placing spring-traps inside the nest-boxes. A small portion of our sample (5% of males and 8% of females) was captured after the laying period had begun using spring-traps in supplementary nest-boxes whose location was changed every second day. Birds belonging to cohorts from 2012 to 2015 (included) were PIT-tagged the first time they were recaptured as adult birds. Cohorts from 2016 to 2019 were PIT-tagged as nestlings. Adults were sexed *de visu* based on clear-cut dimorphic traits (throat feathers and colour of the base of the beak; Hiraldo and Herrera 1974). Once caught, we recorded body mass with a precision digital scale to the nearest 0.1 g, tarsus and beak length with a digital calliper (accuracy = 0.01 mm) and wing length with an end-stop ruler (accuracy = 1 mm). We also quantified plumage spottiness by using a 3-level scoring scale for different parts of the body (head, back, rump, belly and undertail coverts: online resource 3), where 0 corresponded to absence of spots and 2 to large and numerous spots. These scores were all summed up generating a total spottiness variable that ranged from 0 to 10 that was used in the analyses. In addition, we plucked three ornamental throat feathers from each individual (online resource 4) and measured their length in the lab with a digital calliper (accuracy = 0.01 mm). The length of these three feathers was repeatable (*R* = 0.96, SE = 0.003, *p* < 0.001), thus we used the average of the three measurements for analysis.

During each breeding season, we routinely deployed PIT-tag readers (LID-650 decoder, Trovan Ltd, UK) at the nest-boxes to identify the owners and visitors of each nest. PIT-tag readers consisted of an antenna that scanned any PIT-tagged bird that entered a nest-box and a reader, connected to a 12 V battery that stored the records and the time at which they were taken. These readers were deployed throughout the different stages of a breeding event for each nest-box in both first and second broods, which are resumed in three broad stages: (1) pre-laying period, (2) laying-incubation period and (3) chick-rearing period. Readers were installed for 1.5–2 days. In the case of the chick-rearing period, PIT-tag readers were deployed when nestlings were between 6 and 10 days old. The total amount of recorded hours for each year was: 9108 h (2013), 7024 h (2014), 5699 h (2015), 5822 h (2016), 9921 h (2017), 5012 h (2018), 14515 h (2019) and 26973 h (2020). The greater number of recording hours in 2019 and 2020 is due to the installation of solar panels in 30 nest-boxes, which monitored PIT-tagged birds’ activity continuously. The mean number of recording hours per nest-box for each year is available in online resource 5. As a great proportion of adult individuals were PIT-tagged (online resource 6), we were able to determine the owners of each nest-box with a high level of certainty. We used different criteria for each sex to determine nest ownership. In the case of females, we determined ownership with a nocturnal scanning carried out using a long-range transponder-reader (GR250, Trovan) during incubation and with the information from the PIT-tag readers during the chick-rearing period (females detected more than 6 h with at least 1 visit per hour). In the case of males, the owner was determined as the bird that visited the nest most frequently before breeding and during chick feeding. Male ownership status was easily scored for most nest-boxes, although in some nest-boxes, the presence of a male with a low visiting rate was difficult to interpret since it could imply either a low-attending male or a persistent floater in a nest owned by an un-tagged male. We followed the same criteria as with females and we only assigned male ownership when males were detected for 6 or more hours during the time of monitoring with the PIT-tag readers.

Lastly, we considered a bird as a floater when it was detected (through capture sessions or PIT-tag readers) but not recorded breeding in any nest-box throughout the whole breeding season. Due to the existence of some natural holes in our field area, it is possible that a small percentage of our presumed floaters were nesting in natural cavities. To determine whether our criterion for floater status led to a bias in our estimates, we conducted a duplicated analysis (see “Statistical analyses” section) establishing a stricter criterion for floaters: only considering floaters those individuals detected visiting nest-boxes owned by other conspecifics once the chick-rearing period had begun, where floater activity is the highest (Veiga et al. 2012, D. Gil, unpublished data). We did not find a change in the strength nor in the direction of results using this stricter criterion, and thus we adhered to the original criterion in the results of the study. Analyses made with the alternative stricter criterion can be found in the online resources 7, 8 and 9.

Our dataset for adult traits analyses consisted of a total of 280 males and 241 females (online resource 10). In the case of the analysis using nestling predictors, we had data from 276 males and 267 females (online resource 10). Differences in sample size between adult and nestling analyses are due to missing data in some adult measurements.

### Statistical analyses

All analyses were performed in the R language v. 3.6.1 (R Core Team 2020). We ran separate analyses for females and males due to differences in the age of first reproduction. We used the *lme4* package (Bates et al. 2015) to build generalized linear mixed models (GLMMs) with a binomial distribution and a logit link function. We built four binomial GLMMs: two for males and two for females (one for adult traits and one for nestling traits). The dichotomous response variable ‘reproductive status’ had two possible outcomes: floater versus breeder. All analyses were conducted using ‘floater’ as the reference level.

To obtain a robust index of body size, we took the first component of a principal component analysis (PCA) on three morphological traits: beak, wing, and tarsus length. This was done separately for males and females. In the case of males, the first principal component (PC1) had an eigenvalue of 1.22 and explained 40.58% of the variance. All loading scores were positive (tarsus length: 0.66, wing length: 0.54, and beak length: 0.53). In the case of females, PC1 obtained an eigenvalue of 1.29 and explained 42.87% of the variance, with all scores being positive (tarsus length: 0.63, wing length: 0.59, and beak length: 0.51).

Both male and female adult models included body condition, body size and throat feather length as predictors. In the female model of adulthood predictors, we added the degree of spottiness. Since body mass can fluctuate by the time and date of measurement (especially in females due to egg formation), we incorporated these temporal variables in the calculation of body condition. In the case of males, body condition was calculated as the residuals of a linear model in which body mass depended on the time of capture and the body size index (PC1). In the case of females, body condition was calculated as the residuals of the relationship between body mass and body size index and date of measurement. We tested the possibility that effects varied between years by building models that included the interaction of year with each trait (data not shown), but we found no relevant interaction and chose to consider year as a random effect in the models.

In the case of models using early life predictors, ‘reproductive status’ was again used as the binary response variable. Body condition, hatching date and brood size were included as continuous predictors. In the analyses, nestling body condition was calculated as the residuals of a linear model of body mass dependent on tarsus length, corrected by the observer identity. Hatching date was expressed as the relative number of days elapsed between the first hatched egg in the colony and the hatching date of the individual. Brood size corresponded to the number of siblings alive at the moment of nestling measurements. Cohort year was included as a random factor.

For all the analyses we scaled the variables and examined residual plots to check for residual normality and homoscedasticity by using the package *DHARMa* (Hartig 2020). We did not detect multicollinearity (VIF coefficients for all variables in every model were close to 1).

Based on the binomial GLMMs results (see “Results” section), we decided to perform a path analysis to examine the direct and indirect effects of our variables on reproductive status. Path analysis is a valuable tool to calculate the partial correlation coefficients between variables while controlling for the rest of the variables present in the model. Path analysis allows calculating (1) direct effects (path coefficients of arrows directly connecting two variables), (2) indirect effects (calculated as the multiplication of path coefficients of two variables that share an intermediary) and (3) total effects (computed as the sum of direct and indirect effects). We built two-path analyses (one for each sex) using the package *piecewiseSEM* (Lefcheck 2016). Conditional independence claims were evaluated by directed separation tests to determine the goodness of fit of each model (Pearl 2009). The result of these tests is summed in the Fisher’s C statistic (Shipley 2000). Temporal tiers were used to orient the path, as events that happen earlier in life can only be parental nodes of the variables that occur later in life. Moreover, within a tier, we excluded the presence of colliders by calculating the independencies and dependencies of all triplets of variables (Scheines et al. 1994). All variables and their standard errors were scaled to obtain standardized coefficients. For this analysis, we used those individuals in which both nestling and adult measurements were complete, with a final sample size of 261 for males and 231 for females. The model with reproductive status as the response variable was a binomial GLMM with a logit link function while the models for the rest of the variables were linear mixed models with Gaussian distribution. All models included year as a random factor.

## Results

### Adulthood predictors

**Fig. 1:**
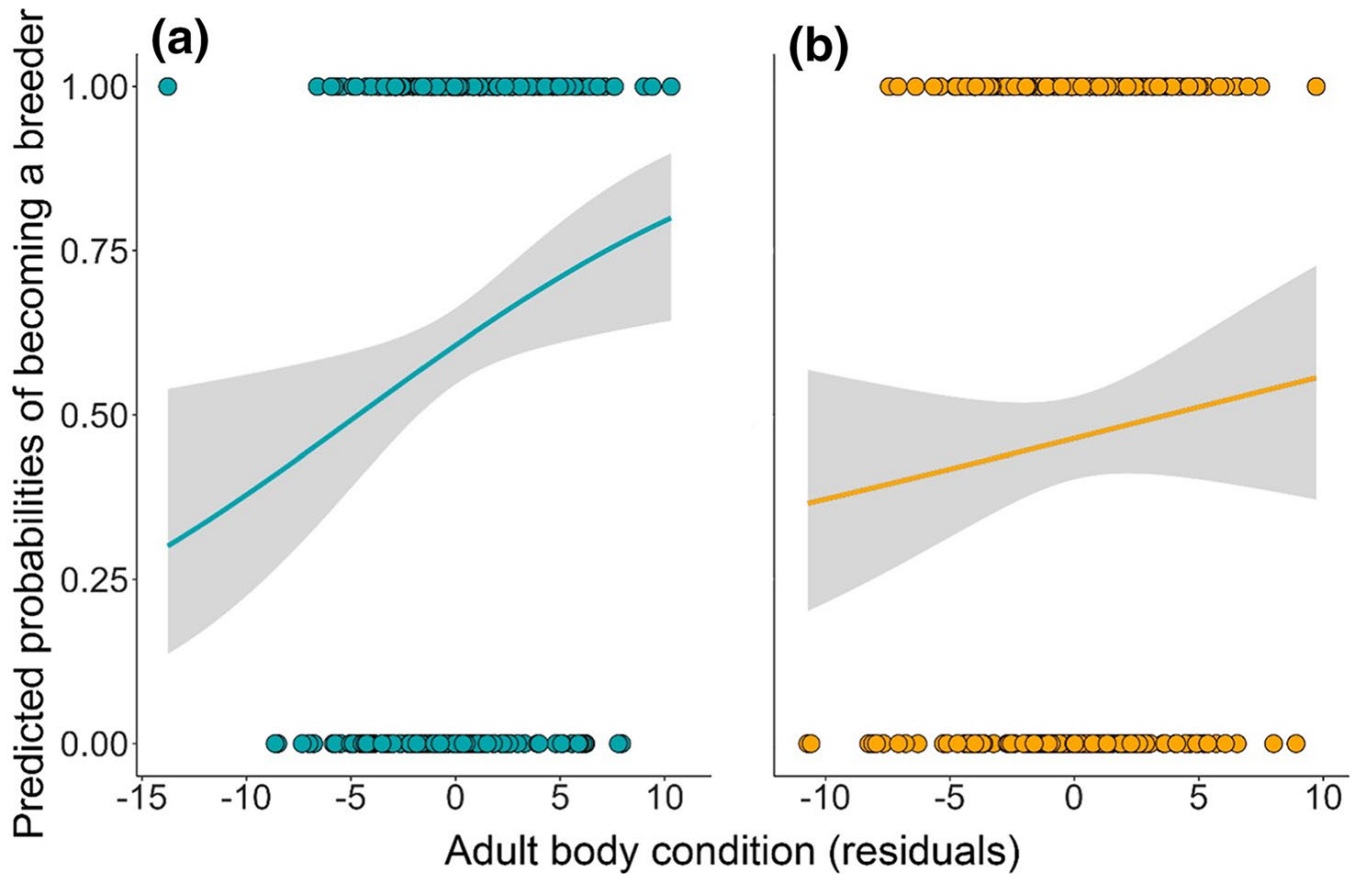
Predicted probabilities, derived from GLMM binomial models with logit as link function, of becoming a breeder in relation to adult body condition for **a** males and **b** females. Body condition is represented by the residuals of body mass regressed on a body size index (PC1). Body condition was corrected by time and date of capture for males and females respectively. Shaded areas around the curve represent 95% CI. Dots represent reproductive status of individuals: 1 = breeders; 0 = floaters

Body condition showed a positive significant association with male reproductive status: increased body condition incremented the likelihood of becoming a breeder (Table 1; Fig. 1a). Neither size nor throat feather length influenced male reproductive status (Table 1).

**Table 1:** GLMMs binomial models with logit as link function for male and female adult starlings to test the influence of body condition, body size (PC1) and ornamentation on the reproductive status in the first reproductive attempt

	Estimates ± SE	*z*	χ^2^	Odds Ratio (CI 95%)	*P* value
**Adult males** (*N* = 280)					
*Initial model*					
Fixed effects					
Intercept	0.434 ± 0.124	3.49			
**Body condition**	**0.309** ± **0.129**	**2.39**	**5.96**	**1.36 (1.06–1.75)**	**0.015**
Size	0.137 ± 0.125	1.10	1.21	1.15 (0.90–1.46)	0.271
Feather length	0.114 ± 0.128	0.89	0.80	1.12 (0.87–1.44)	0.371
*Final model*					
Fixed effects					
Intercept	0.431 ± 0.124	3.48			
**Body condition**	**0.324** ± **0.128**	**2.52**	**6.67**	**1.38 (1.07–1.78)**	**0.009**

	Estimates ± SE	*z*	χ^2^	Odds Ratio (CI 95%)	*P* value
**Adult females** (*N* = 241)					
*Initial model*					
Fixed effects					
Intercept	− 0.203 ± 0.215	− 0.94			
Body condition	0.157 ± 0.137	1.15	1.32	1.17 (0.89–1.53)	0.250
Size	0.199 ± 0.138	1.20	2.11	1.22 (0.93–1.60)	0.146
Feather length	− 0.199 ± 0.156	− 1.28	1.72	0.82 (0.60–1.11)	0.189
Spottiness	− 0.190 ± 0.141	− 1.35	1.84	0.83 (0.63–1.09)	0.175
*Final model*					
Intercept	− 0.208 ± 0.225	− 0.92			

In the case of females, an additional phenotypical variable, spottiness, was included in the models. The dependent variable (reproductive status) is coded so that 1 = breeder and 0 = floater. Floater status is used as the reference level. We report the variable estimates calculated from an initial model (model including all variables) and a final model (model retaining only significant terms). In case there were no significant terms, the intercept is shown. All variables are scaled. Significant results are presented in bold

In the case of females, body condition was not associated with reproductive status (Table 1; Fig. 1b). Similarly, neither did the rest of examined morphological traits (Table 1).

### Nestling predictors

**Fig. 2:**
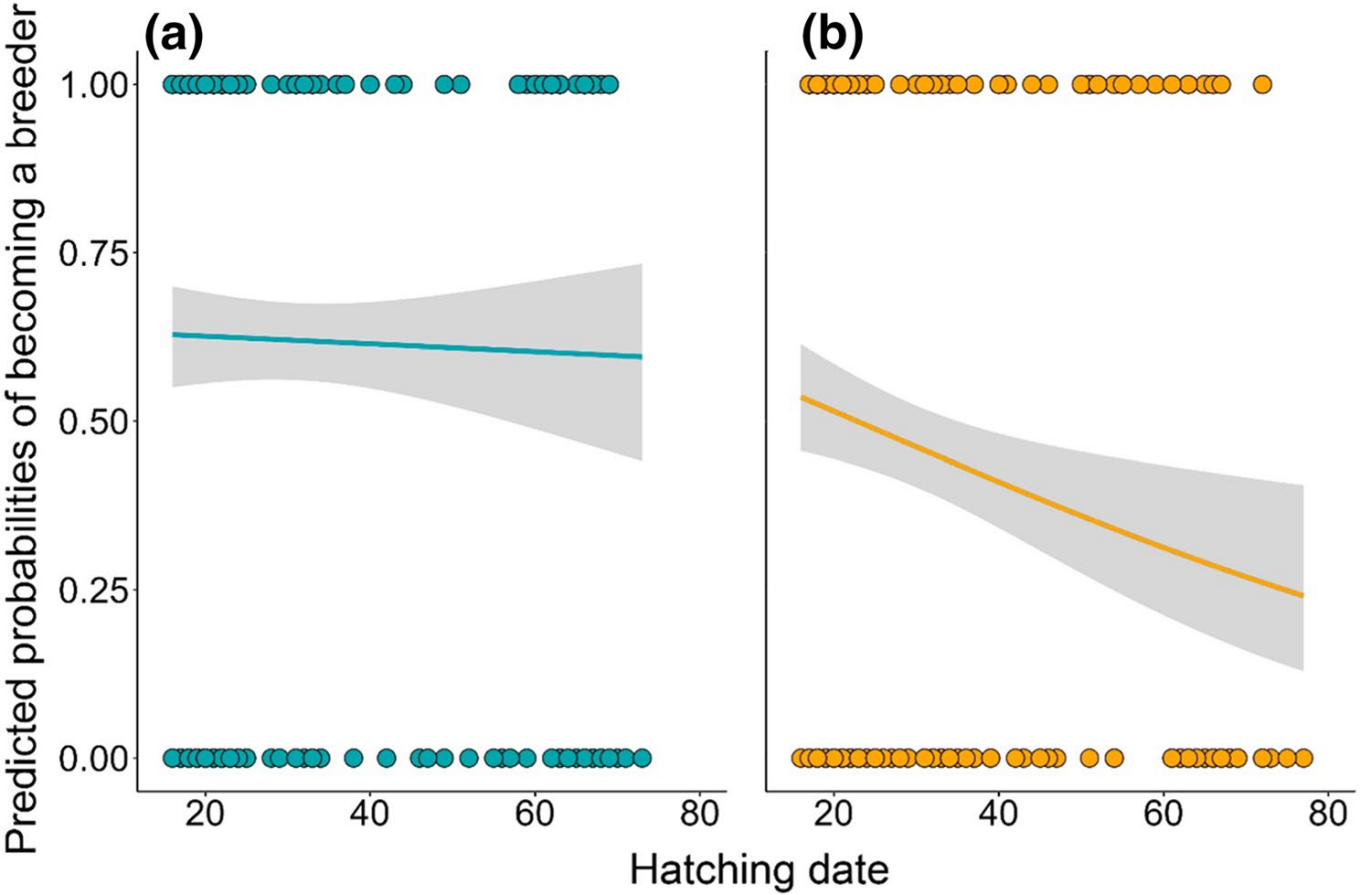
Predicted probabilities, derived from GLMM binomial models with logit as link function, of becoming a breeder in relation to hatching date for **a** males and **b** females. Hatching date is expressed as the hatching day of an individual relative to the first hatched chick in the breeding season. Shaded areas the curve around represent 95% CI. Dots represent reproductive status of individuals: 1 = breeders; 0 = floaters

When considering nestling condition and proxies of early life environment (hatching date and brood size) as predictors of reproductive status, we found the same association as for adult males: males that were in better condition as nestlings were also more likely to become breeders (Table 2), with the rest of variables, hatching date (Fig. 2a) and brood size, showing no association with reproductive status later in life (Table 2).

**Table 2:** GLMM binomial models with logit as link function for male and female nestling starlings to test the influence of body condition, hatching date and brood size on the reproductive status in the first reproductive attempt

	Estimates ± SE	*z*	χ^2^	Odds Ratio (CI 95%)	*P* value
**Nestling males** (*N* = 276)					
*Initial model*					
Fixed effects					
Intercept	0.503 ± 0.127	3.98			
**Body condition**	**0.401 ± 0.136**	**2.94**	**9.09**	**1.49 (1.14–1.95)**	**0.003**
Hatching date	0.105 ± 0.135	0.78	0.61	1.11 (0.85–1.45)	0.435
Brood size	0.139 ± 0.129	1.08	1.18	1.15 (0.89–1.48)	0.278
*Final model*					
Fixed effects					
Intercept	0.502 ± 0.126	3.98			
**Body condition**	**0.369** ± **0.129**	**2.86**	**8.54**	**1.45 (1.12–1.86)**	**0.003**

	Estimates ± SE	*z*	χ^2^	Odds Ratio (CI 95%)	*P* value
**Nestling females** (*N* = 267)					
*Initial model*					
Fixed effects					
Intercept	− 0.237 ± 0.244	− 0.97			
Body condition	− 0.004 ± 0.145	− 0.03	0.01	1.00 (0.75–1.32)	0.977
**Hatching date**	− **0.336 ± 0.158**	− **2.13**	**4.69**	**0.71 (0.52–0.97)**	**0.030**
Brood size	0.104 ± 0.137	0.76	0.59	1.11 (0.84–1.45)	0.446
*Final model*					
Fixed effects					
Intercept	− 0.239 ± 0.243	− 0.98			
**Hatching date**	− **0.363 ± 0.140**	− **2.59**	**7.11**	**0.69 (0.53–0.92)**	**0.008**

The dependent variable (reproductive status) is coded so that 1 = breeder and 0 = floater. Floater status is used as the reference level. We report the variable estimates calculated from an initial model (model including all variables) and a final model (model retaining only significant terms). In case there were no significant terms, the intercept is shown. All variables are scaled. Significant results are presented in bold

For females, we detected a negative association between hatching date and reproductive status: late hatched females had a lower likelihood of becoming breeders the next season (Table 2; Fig. 2b). Neither body condition nor brood size influenced reproductive status in females (Table 2).

### Path analysis

**Fig. 3:**
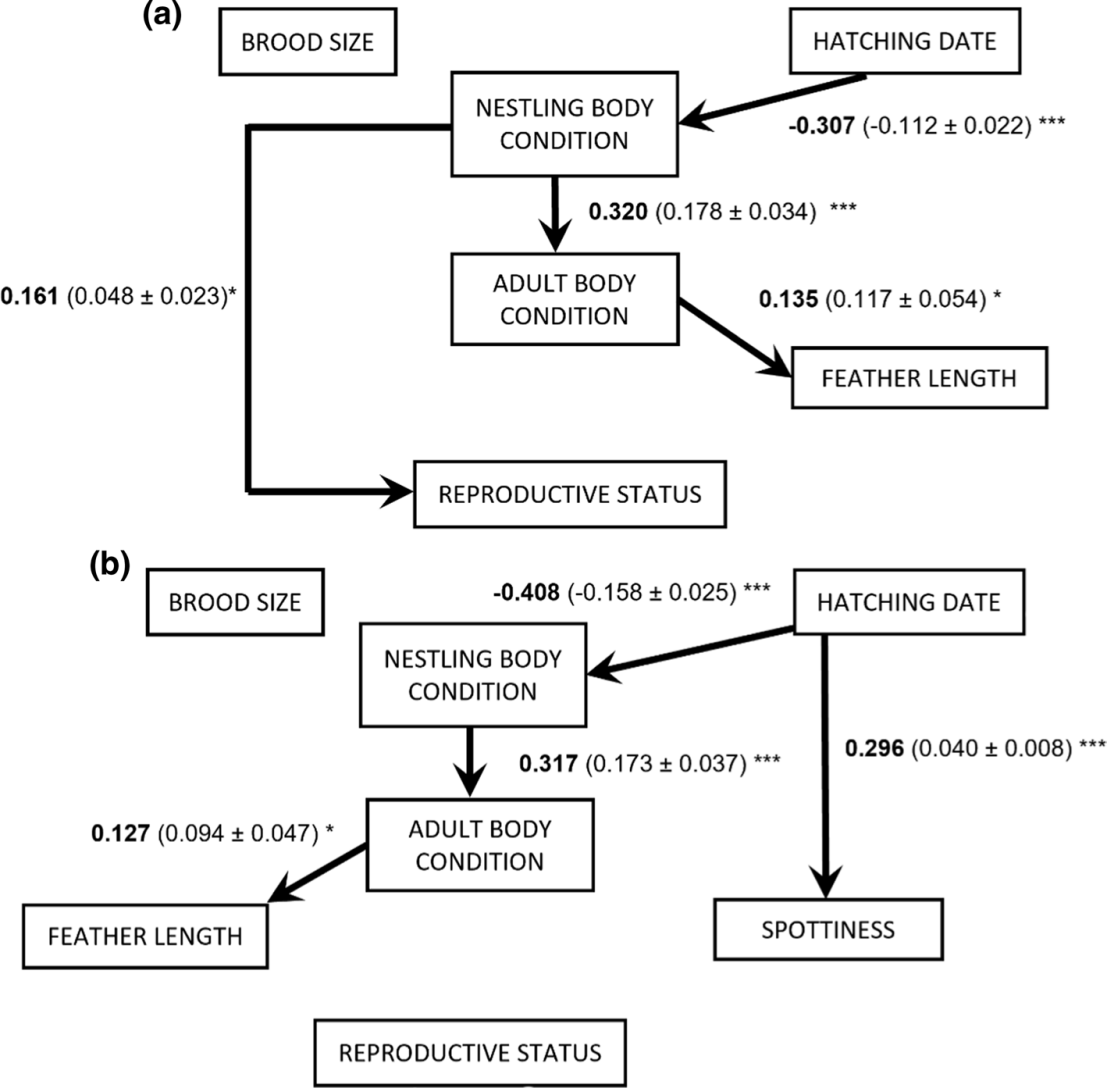
Path analysis diagrams for **a** males and **b** females. Only significant relationships are shown (* = *P* < 0.05, ** = 0.01 < *P* < 0.05, *** = *P* < 0.01). Standardized path coefficients are presented with their corresponding unstandardized coefficient ± standard error in parenthesis

Both male and female path analysis satisfied the conditional claims (Male path: C_6_ = 3.01, *P* = 0.80, *N* = 261, Fig. 3a; Female path: C_14_ = 14.24, *P* = 0.43, *N* = 231, Fig. 3b), showing that the structure of the paths was supported by our dataset.

In the case of male models, only nestling body condition had a direct causal effect on reproductive status (Fig. 3a). Path analysis showed that hatching date was indirectly and negatively linked to reproductive status via nestling body condition (indirect effect = − 0.307 × 0.161 = 0.05). Neither brood size, ornamentation nor adult body condition was linked to reproductive status, neither directly nor indirectly. Independently of their effect on reproductive status, we found that nestling body condition was positively associated with adult body condition, and this, in turn, was positively linked with a secondary sexual character, feather length.

In the case of females (Fig. 3b), we only detected a marginally significant (*P* = 0.057) direct and negative effect of hatching date on reproductive status, i.e., females born earlier in the season were more likely to become a breeder as 1-year-olds. We did not detect any other direct or indirect relationship between our predictors and reproductive status in females. When looking at the association between our explanatory variables, we found parallel results to those of males: hatching date was also negatively associated, but with a stronger effect, with nestling body condition, and this had also a positive link with adult body condition (indirect effect = − 0.408 × 0.317 = − 0.129). We also detected a positive and direct relationship between adult body condition and feather length in females. Lastly, females that were born later in the season showed a higher degree of spottiness in their first purported breeding year.

## Discussion

Floaters are a common but often neglected component of avian populations (Lenda et al. 2012). The lack of information about floaters may result in biased estimates of population dynamics and sexual selection in the wild (Penteriani et al. 2011; Moreno 2016), in particular if they differ phenotypically from breeders. In this study, we examined if a series of adult morphological traits and early life conditions was associated with reproductive status (breeder vs. floater) in the spotless starling, a secondary hole-nesting passerine. Our results revealed important differences in predictors of the reproductive status between males and females.

While body condition was not associated with reproductive status in females, it strongly influenced the likelihood of becoming a breeder in the case of males. This suggests male competition for breeding resources (i.e. females, nest sites), within a process of intrasexual selection (Andersson 1994). Our finding is expected given the male-biased sexual dimorphism in size of this species (Cordero et al. 2001), the delayed age of first reproduction in males (Veiga et al. 2012; D. Gil, unpublished data) and the occurrence of polygyny. Males in better condition were more likely to own a nest, possibly reflecting greater capabilities for acquiring and defending resources. Despite not finding an association of body condition with reproductive status in the case of females, female intrasexual competition for nests also occurs (e.g. female-female fights and casualties are often observed). Our data thus suggests that female intrasexual competition is less intense than in males and unrelated to body condition. Previous studies show contrasting evidence in relation to the role of body size or body condition in floater status (Moreno 2016). While some studies also found that male and female floaters were often smaller or in worse body condition than breeders (Sandell and Diemer 1999; Lozano 1994; Emlen and Wrege 2004), others did not (Peer et al. 2000; Fedy and Stutchbury 2004; Sergio et al. 2009). This variety of results suggests that the link between condition and reproductive status likely may be influenced by species ecological and biological requirements and differences in population attributes (e.g. density), etc. (Sergio et al. 2009).

We consider that the most plausible interpretation of our data is that male floaters in bad condition have a low capacity to acquire a nest-box. Alternatively, these birds could be following a strategy: by delaying breeding they might increase the probability of future reproduction. However, this is unlikely given that only 12% of males reproduce for the first time as 3 years old or older (online resource 1 and online resource 2). Thus, it seems that waiting to reproduce and queuing for an available nest site is a suboptimal strategy for males. It is possible that floaters do reap some benefits from alternative mating strategies, although the relatively low level of EPP in the colony (7% of young: Celis et al. 2021), suggests that this is a comparatively poorer strategy.

The case of females, though similar to males in terms of the percentage of individuals that remain floaters, presents an additional nuance. In contrast to males, the percentage of females that breed for the first time after their first year is relatively high (30%: online resource 1). Thus, it seems that waiting to reproduce is less penalized in females than in males. Like males, female alternative reproductive strategies (conspecific brood parasitism or quasiparasitism, Monclús et al. 2017) seem to provide a comparatively lower fitness than owning a nest (7% and 1% of young respectively: Celis et al. 2021). Nevertheless, the proportion of nests containing parasite nestlings increased as the colony aged and the population grew (Celis et al. 2021), suggesting that the increase of floaters was responsible for this increase in parasitism. Although we still lack an estimation of the true reproductive success of floaters through alternative breeding strategies, our data suggest that floating in this population responds to a “best of a bad job” strategy.

Interestingly, the positive link between male adult body condition and the probability of becoming a breeder disappeared when the early part of the life cycle of the individuals (i.e. nestling stage: brood size, hatching date and nestling body condition) was incorporated into the picture (Fig. 3a), showing that the reproductive status attained in adulthood is mainly explained by nestling condition. The relevance of this effect is surprising given the extended time gap but adds support to previous studies that show that early life conditions can have long-lasting effects on birds (Lindström 1999; Monaghan 2008; Szász et al. 2017; Saino et al. 2018). This result is in line with other studies that show a connection between early life and related life-history traits such as survival, natal dispersal, or recruitment probability (Verhulst et al. 1997; Reid et al. 2003; van de Pol et al. 2006; Becker and Bradley 2007; Azpillaga et al. 2018).

In the case of females, early hatching birds were also more likely to recruit as breeders than floaters. Cordero et al. (2001) reported that hatching date was also negatively related to local recruitment in females in the same species. In males, although hatching date indirectly influenced reproductive status through nestling condition, its effect was small and superseded by that of condition. This difference between sexes in the effect of hatching date suggests that poor-breeding conditions experienced in late broods are particularly detrimental for females. This could be due to males being better at competing with female siblings in the nest. In line with this result, we have found in this population that females suffer more strongly the consequences of enlarged brood sizes than males (Gil et al. 2019). Thus, it is possible that males, being larger than females already at the nestling stage (Muriel et al. 2021), are less negatively affected in late broods, when conditions are harsher and brood reductions are more frequent. An alternative possibility is that the effect of hatching date diminishes with time and, since males are studied a year later than females, this time difference buffers the effect of the hatching date. However, given that we do find a long-term effect of body condition, this alternative explanation is not very likely. We have no clear explanation for the causal link between female hatching late in the season and becoming a floater, although several possibilities can be envisaged. Breeding conditions decrease with advancing season in this population (Gil et al. 2008; Muriel et al. 2015), and this suggests that late hatching birds encounter a more hostile environment when they become independent, with a drier weather and fewer insects. This may reduce the capacity of late-hatching females to obtain resources, in particular, if they are excluded from advantageous positions in winter flocks, as has been shown in other species (Verboven and Visser 1998).

Contrary to our predictions, throat feather length showed no association with reproductive status neither in males nor in females, a result that highly contrasts with the common finding of territory owners of other species exhibiting larger (Emlen and Wrege 2004; Johnson et al. 2000) or more intense ornamentation (Velando et al. 2001; Blas et al. 2013) than floaters. The surprising lack of influence on reproductive status suggests that, in the spotless starling, feather length either does not provide a reliable assessment of fighting capacity beyond the first year, or that fights for nest-boxes are settled by direct assessment of body condition or other ornaments. However, our path analysis shows that feather length is positively associated with body condition in both sexes (Fig. 3a, b). It is possible that this condition and age-dependent ornament may be more relevant in the context of mate choice (Aparicio et al. 2001; Komdeur et al. 2005) than in intrasexual competition for breeding resources. Although inter-and intrasexual sexual selection often share the same target traits (Andersson 1994), it has been shown that some traits can become superfluous as a result of adaptive flexibility in mate choice criteria by females (Chaine and Lyon 2008). In addition, feather length is only a reliable index of age for 1-year old vs. older birds (D. Gil, unpublished data). Since our study focuses on 2-year-old males, it is possible that we may have missed the role that this trait may have as a reliable index of male age in younger birds.

To conclude, we found a strong association between body condition and the probability of becoming a breeder in male spotless starlings, suggesting a link between individual quality and access to breeding. Moreover, nestling body condition was found to influence male reproductive status directly. This pattern was absent in females, suggesting a more relaxed regime of intrasexual competition for this sex in the access for nesting sites. However, early-born females had an increased likelihood of breeding as 1-year-olds. Our results highlight the influence that early life may have on the future breeding status and shed light on the phenotype of floating individuals. Next steps would require the examination of life-history traits such as survival and reproductive success to understand floating behaviour and to evaluate its consequences on fitness.

## Supplementary Information

Below is the link to the electronic supplementary material.

Supplementary file1 (PDF 569 KB)

## Data Availability

Data used for this manuscript is available in the Figshare repository, at the permanent link: 10.6084/m9.figshare.19705033.
