# Supplementary material for: Sexual differences in phenotypical predictors of floating status: body condition influences male but not female reproductive status in a wild passerine
Source: Oecologia. 2022 May 12;199(1):79–90. doi: 10.1007/s00442-022-05180-1 (PMC9119866; doi:10.1007/s00442-022-05180-1)
Supplement: Supplementary file 1 — Supplementary file1 (PDF 569 KB) [file 442_2022_5180_MOESM1_ESM.pdf]

Sexual differences in phenotypical predictors of floating status: body condition influences male but not female reproductive status in a wild passerine.

Iraida Redondo[1]*, Lorenzo Pérez- Rodríguez[2], Raquel Monclús[3], Jaime Muriel[4] & Diego Gil[1]

Supporting Information:

This Word file contains supplementary material:

- Supplementary Figures
- Supplementary Tables.

[1]Departamento de Ecología Evolutiva, Museo Nacional de Ciencias Naturales, MNCN (CSIC), José Gutiérrez Abascal 2, E-28006 Madrid, Spain.

[2]Instituto de Investigación en Recursos Cinegéticos, IREC (CSIC, UCLM, JCCM), Ronda de Toledo 12, 13005, Ciudad Real, Spain

[3]Laboratoire d'Ethologie Expérimentale et Comparée UR 4443, Université Sorbonne Paris Nord, 93430, Villetaneuse, France

[4]Department of Zoology, Faculty of Sciences, University of Granada, E-18071, Granada, Spain.

* Corresponding author: Iraida Redondo (iraidaredondogar@gmail.com)

Online resource 1. Age of first reproduction (AFR) of two cohorts PIT-tagged as nestlings monitored in our colony of spotless starlings. The number of individuals in these tables correspond to detected starlings in our nest-box colony. We denominated as 'Permanent floaters' those individuals detected but never found breeding in any of our nest boxes throughout the years.

Males

Cohort	Permanent floaters	AFR = 1	AFR = 2	AFR = 3	AFR = 4	Total
2016	33	3	32	10	1	79
2017	80	4	30	16	2	132
Total (percentage from the total)	113 (54%)	7 (3%)	62 (29%)	26 (12%)	3 (1%)	211

Females

Cohort	Permanent floaters	AFR = 1	AFR = 2	AFR = 3	AFR = 4	Total
2016	23	15	23	1	0	62
2017	34	40	27	1	1	103
Total (percentage from the total)	57 (35%)	55 (33%)	50 (30%)	2 (1%)	1 (1%)	165

Online resource 2. Barplot representing the age of first reproduction (AFR) across two cohorts of spotless starlings (Online resource 1).

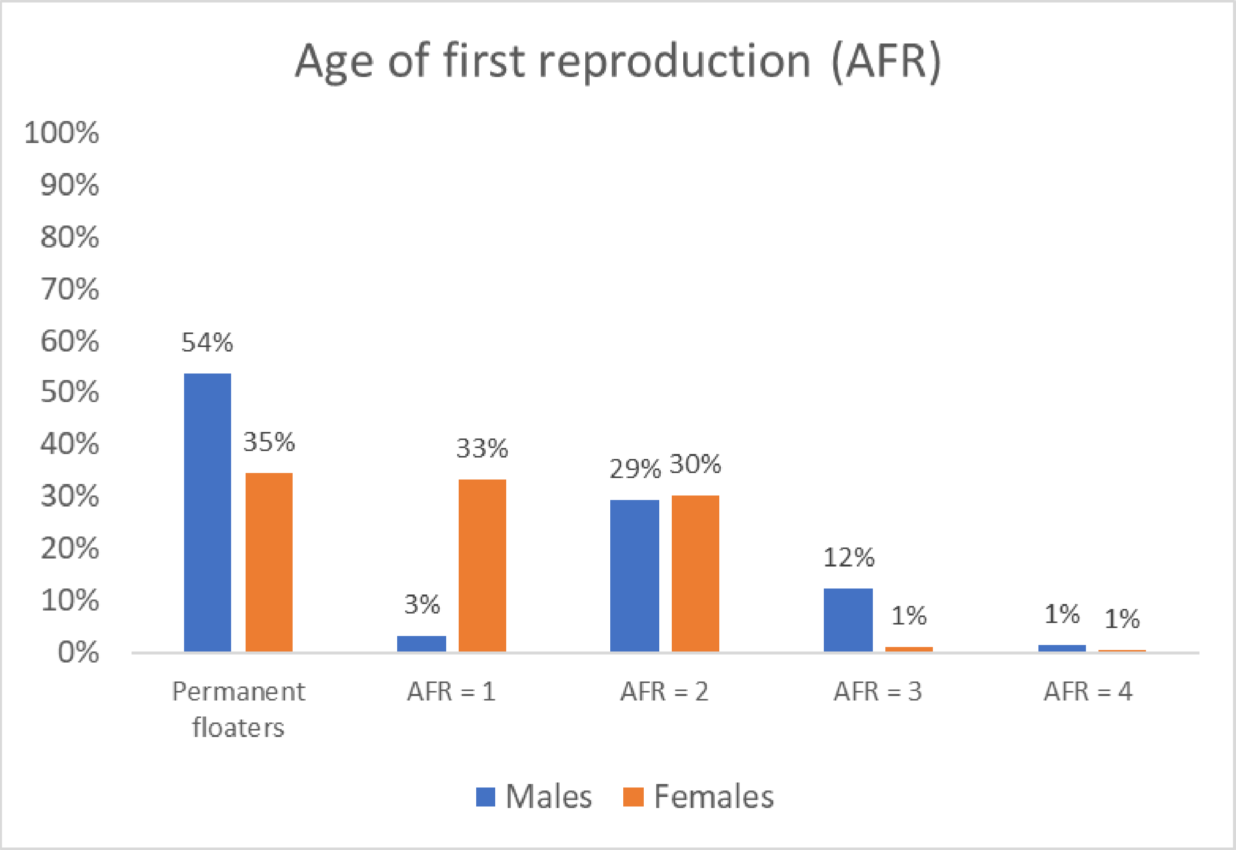

Online resource 3. Criteria followed to quantify the amount of spottiness present along the body of spotless starlings. The degree of spottiness is calculated as the sum of the scores assigned for each body part.

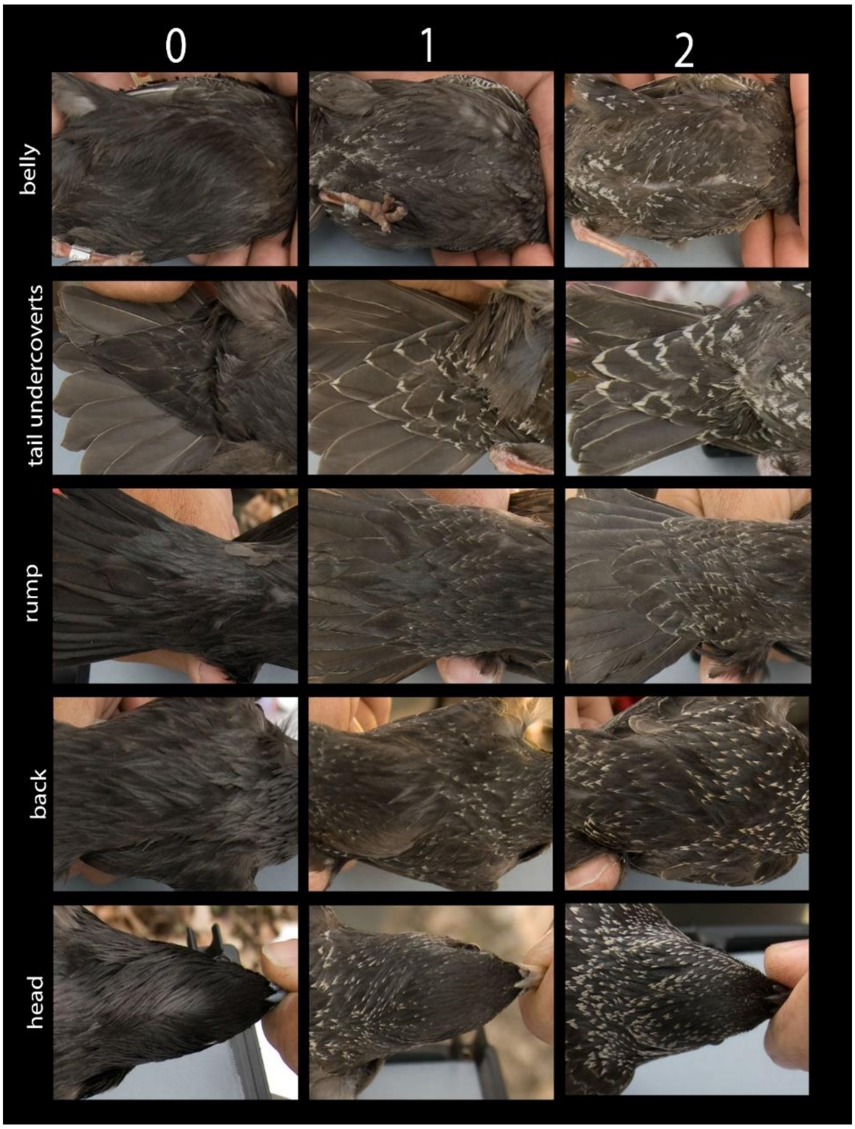

Online resource 4. **(a)** Example of collection of ornamental feathers showing an adult male spotless starling. **(b)** Comparison of the shape and length of throat ornamental feathers in female and male spotless starlings. Picture credit: Lorenzo Pérez-Rodríguez

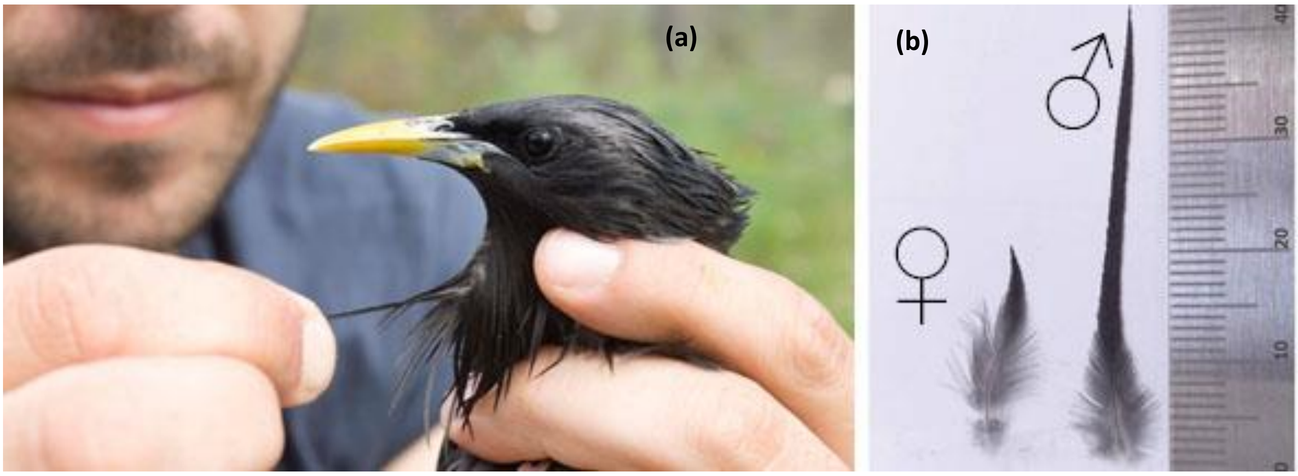

Online resource 5. Mean hours of PIT-tagged reader deployment per nest box in each year of study and its standard deviation.

**YEAR**	Nest boxes monitored	Mean (hours/nest box)	Std. Dev.	Total recording hours
2013	245	37.18	18.24	9108
2014	244	28.91	17	7024
2015	226	25.22	17.47	5699
2016	240	24.26	19.86	5822
2017	246	40.33	21.88	9921
2018	244	20.9	20.46	5012
2019	246	59	95.11	14515
2020	243	111	136.56	26973

Online resource 6. Number and percentage of identified females and males in successful breeding events in our colony per year of study. We considered 'successful' those breeding attempts with at least one chick fledged.

**YEAR**	Known female (%)	Known male (%)	Total of successful breeding events
2012	208 (75)	187 (67)	278
2013	232 (75)	253 (82)	309
2014	277 (69)	267 (66)	404
2015	274 (68)	210 (52)	402
2016	300 (83)	251 (70)	360
2017	310 (83)	284 (76)	372
2018	314 (89)	256 (73)	353
2019	291 (88)	243 (74)	329
2020	325 (88)	279 (75)	371

Online resource 7. This table resumes the number of breeders and floaters individuals from each cohort that were considered for the restrictive analysis. In this case, we applied a more restrictive criterion for including floaters (i.e. we considered floaters only those individuals detected from the rearing chick period onwards). Due to their earlier age of first reproduction, females were captured as 1-year-olds (cohort year + 1) whereas males considered in this study were captured as 2-year-olds (cohort year + 2). Note that N differs between adults and nestlings due to missing data in some individuals.

		Male (nestlings)		Male (2-year-old)		Female (nestlings)		Female (1-year-old)	
Cohort	Total nestlings marked	Territorials	Floaters	Territorials	Floaters	Territorials	Floaters	Territorials	Floaters
2012	917	18	3	16	4	15	4	14	3
2013	1137	23	12	21	6	19	2	11	2
2014	1598	31	3	26	7	25	15	24	14
2015	1427	26	8	25	14	8	6	7	4
2016	1246	29	11	23	13	2	10	2	9
2017	1336	17	7	30	6	17	8	20	9
2018	1189	26	17	28	13	22	4	22	4
2019	1241					14	13	11	8
Total per status		170	61	169	63	122	62	111	53
Total per sex		231		232		184		164	

Online resource 8. GLMMs binomial models with logit as link function for male and female adult spotless starlings to test the influence of body condition, body size (PC1) and ornamentation on the reproductive status of spotless starlings in their first potential reproduction event. In the case of females, an additional phenotypical variable, spottiness, was included in the models. The dependent variable (reproductive status) is coded so that 0 = floater and 1 = breeder. Floater status is used as the reference level. We report the variable estimates calculated from an initial model (model including all variables) and a final model (model retaining only significant terms. In case there were no significant terms, the intercept is shown). All variables are scaled. This corresponds to the results using the most restrictive criterion in assigning floating status (see Online resource 7 legend). Significant results are presented in bold.

	Estimates ± SE	z	χ^2	Odds Ratio (CI 95%)	P-value
Adult males (n = 232)					
**Initial model**					
Fixed effects					
Intercept	1.049 ± 0.189	5.55			
**Body condition**	0.358 ± 0.157	**2.28**	**5.45**	**1.43 (1.05 - 1.95)**	**0.020**
Size	0.021 ± 0.149	0.14	0.02	1.02 (0.76 - 1.37)	0.885
Feather length	0.049 ± 0.156	0.31	0.10	1.05 (0.77 – 1.43)	0.755
**Final model**					
Fixed effects					
Intercept	1.046 ± 0.187	5.60			
**Body condition**	**0.367 ± 0.155**	**2.36**	**5.84**	**1.44 (1.06 – 1.96)**	**0.016**
Adult females (n = 164)					
**Initial model**					
Fixed effects					
Intercept	0.787 ± 0.283	2.78			
Body condition	0.337 ± 0.183	1.84	3.49	1.40 (0.98 – 2.00)	0.062
**Size**	**0.358 ± 0.184**	**1.95**	**3.90**	**1.43 (0.99 – 2.05)**	**0.048**
Feather length	-0.188 ± 0.193	-0.98	0.97	0.83 (0.57 - 1.21)	0.324
Spottiness	-0.084 ± 0.190	-0.44	0.20	0.92 (0.63 - 1.33)	0.657
**Final model**					

Fixed effects					
Intercept	0.764 ± 0.299	2.55			
Size	0.322 ± 0.179	1.80	3.3	1.37 (0.97 – 1.96)	0.070

Online resource 9. GLMMs binomial models with logit as link function for male and female nestling spotless starlings to test the influence of body condition, hatching date and brood size on the reproductive status of spotless starlings in their first potential reproduction event The dependent variable (reproductive status) is coded so that 0 = floater and 1 = breeder. Floater status is used as the reference level. We report the variable estimates calculated from an initial model (model including all variables) and a final model (model retaining only significant terms. In case there were no significant terms, the intercept is shown). All variables are scaled. This corresponds to the results using the most restrictive criterion in assigning floating status (see Online resource 7 legend). Significant results are presented in bold.

	**Estimates ± SE**	**z**	**χ^2**	**Odds Ratio (CI 95%)**	**P-value**
Nestling males					
(n = 231)					
Fixed effect					
**Initial model**					
Intercept	1.110 ± 0.195	5.66			
**Body condition**	**0.358 ± 0.158**	**2.26**	**5.25**	**1.43 (1.05 - 1.95)**	**0.02**
Hatching date	0.010 ± 0.162	0.06	0.00	1.00 (0.73 - 1.39)	0.951
Brood size	0.261 ± 0.163	1.60	2.57	1.30 (0.94 - 1.79)	0.109
**Final model**					
Intercept	1.111 ± 0.216	5.13			
**Body condition**	**0.361 ± 0.153**	**2.36**	**5.74**	**1.43 (1.06 – 1.94)**	**0.017**
Nestling females					
(n = 184)					
**Initial model**					
**Fixed effects**					
Intercept	0.721 ± 0.342	2.11			
Body condition	0.001 ± 0.183	0.00	0.00	1.00 (0.70 - 1.43)	0.998
**Hatching date**	**-0.485 ± 0.198**	**-2.44**	**6.07**	**0.62 (0.42 – 0.91)**	**0.014**
Brood size	0.084 ± 0.188	0.45	0.20	1.09 (0.75 - 1.58)	0.657
**Final model**					

**Fixed effects**					
Intercept	0.720 ± 0.334	2.15			
**Hatching date**	**-0.509 ± 0.173**	**-2.94**	**8.90**	**0.60 (0.42 – 0.84)**	**0.003**

Online resource 10. This table resumes the number of breeders and floaters individuals from each cohort that were considered in the analysis for the main manuscript. For each year, it shows the total number of marked nestlings in our colony and the number of individuals captured as adults to conduct this study and their reproductive status that year. Due to their earlier age of first reproduction, females were captured as 1-year-olds (cohort year + 1) whereas males considered in this study were captured as 2-year olds (cohort year + 2). Note that N differs between adults and nestlings due to missing data in some individuals.

		Male (nestlings)		Male (2-year-old)		Female (nestlings)		Female (1-year-old)	
Cohort	Total nestlings marked	Territorials	Floaters	Territorials	Floaters	Territorials	Floaters	Territorials	Floaters
2012	917	18	9	16	10	15	11	14	10
2013	1137	23	13	21	14	19	11	11	10
2014	1598	31	14	26	15	25	24	24	22
2015	1427	26	17	25	18	9	20	8	16
2016	1246	30	18	23	24	2	21	2	20
2017	1336	17	14	30	12	17	20	20	22
2018	1189	26	20	28	18	22	16	22	15
2019	1241					14	21	11	14
Total per status		171	105	169	111	123	144	112	129
Total per sex		276		280		267		241	
